# Supplementary material for: Genome Size Variation in Sesamum indicum L. Germplasm from Niger
Source: Genes (Basel). 2024 May 29;15(6):711. doi: 10.3390/genes15060711 (PMC11203198; doi:10.3390/genes15060711)
Supplement: Supplementary file 1 [file genes-15-00711-s001.zip › Supplementary data/Supplementary Figures.pdf]

Supplementary Figures

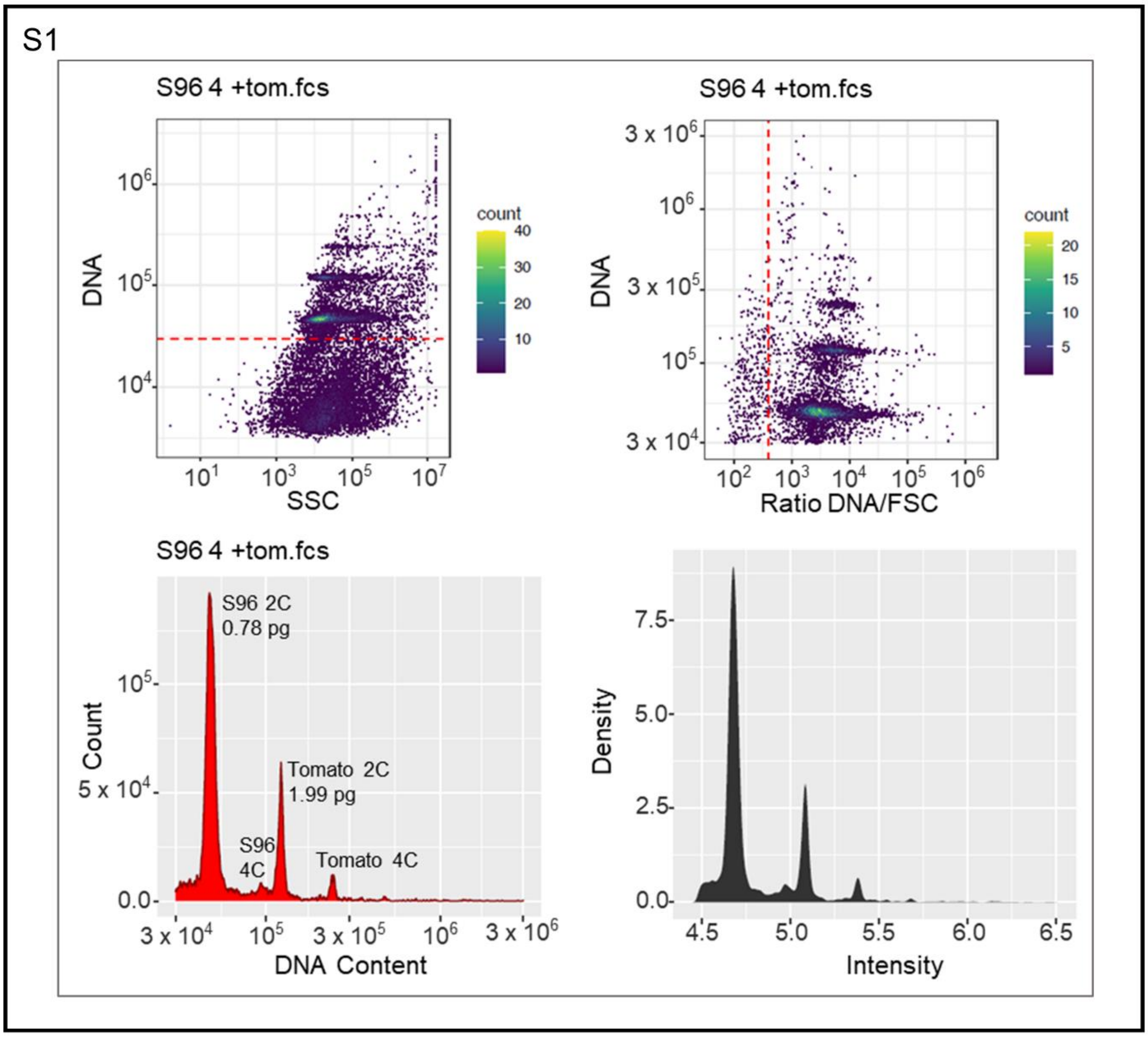

Figure S1. Flow cytometry density plots (top panel) and histograms (bottom panel) for the smallest GS (S96) among the Niger accessions.

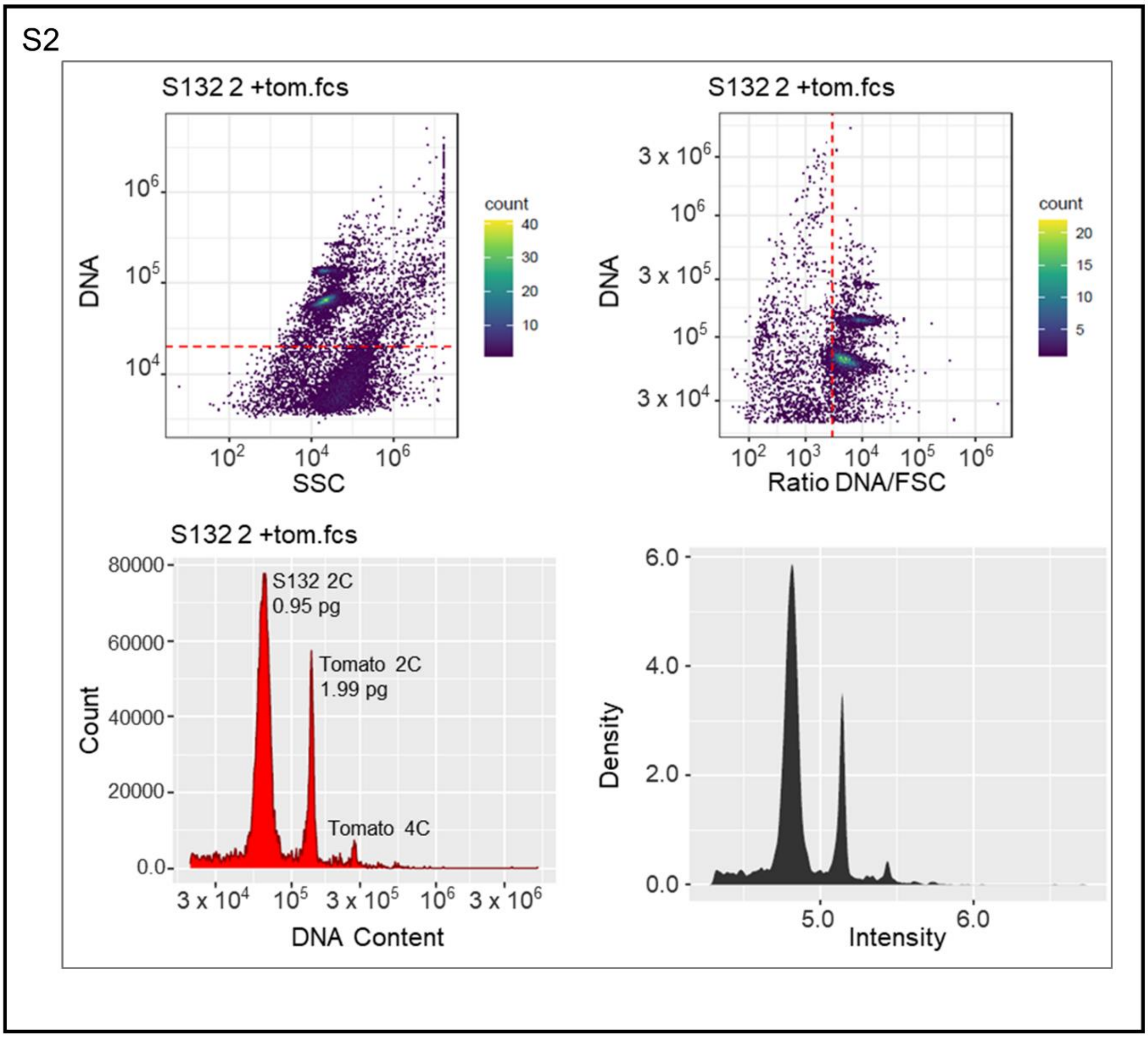

Figure S2. Flow cytometry density plots (upper panel) and histograms (lower panel) for the largest GS (S132) among the Niger accessions.

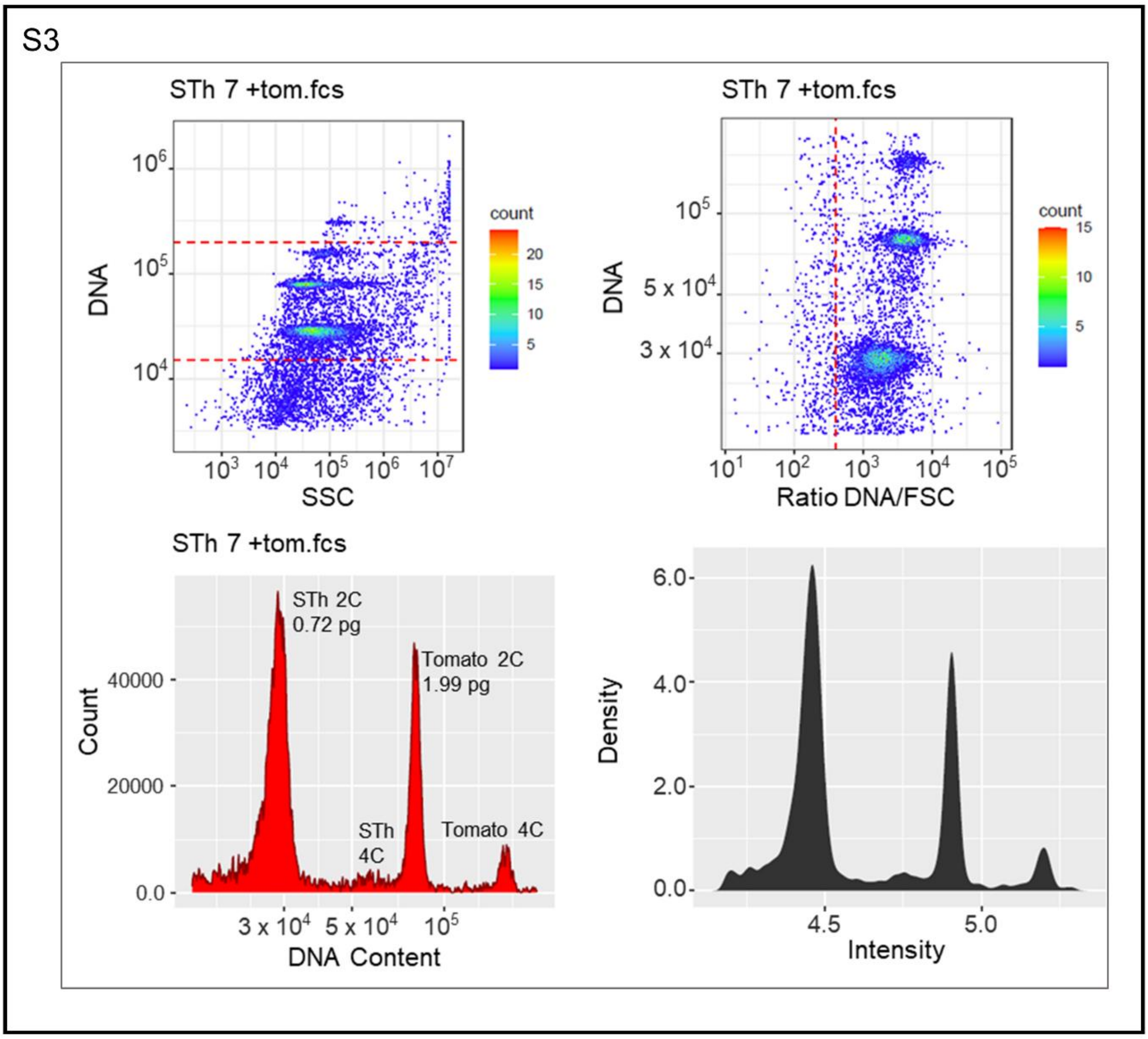

Figure S3. Flow cytometry density plots (top panel) and histograms (bottom panel) for the smallest GS of the Tai STh accession.
