# Supplementary material for: Genome Size Variation in Sesamum indicum L. Germplasm from Niger
Source: Genes (Basel). 2024 May 29;15(6):711. doi: 10.3390/genes15060711 (PMC11203198; doi:10.3390/genes15060711)
Supplement: Supplementary file 1 [file genes-15-00711-s001.zip › Supplementary data/Supplementary Tables.pdf]

Supplementary Tables

Table S1. Descriptive statistical variables of sesame accessions Genome size. Mean 2C value, Standard Deviation (SD), Minimum (Min), First Quartile (Q1), Median, Third Quartile (Q3) and Maximum (Max) 2C values in pg for each accession. n: number of individuals assessed.

Accession	n	Mean 2C value	S.D.	Min	Q1	Median	Q3	Max
**S1**	5	0.87	0.008	0.86	0.87	0.87	0.88	0.88
**S2**	5	0.87	0.012	0.86	0.86	0.87	0.87	0.89
**S3**	5	0.85	0.011	0.83	0.84	0.85	0.85	0.86
**S4**	5	0.84	0.008	0.83	0.83	0.84	0.84	0.85
**S7**	5	0.87	0.013	0.86	0.86	0.87	0.88	0.89
**S8**	5	0.88	0.02	0.86	0.87	0.87	0.89	0.91
**S11**	5	0.84	0.025	0.82	0.82	0.83	0.85	0.88
**S12**	5	0.90	0.031	0.87	0.87	0.90	0.92	0.94
**S13**	5	0.91	0.008	0.90	0.91	0.91	0.92	0.92
**S14a**	5	0.88	0.011	0.86	0.87	0.88	0.88	0.89
**S15**	5	0.80	0.007	0.79	0.80	0.80	0.80	0.81
**S16**	5	0.87	0.018	0.86	0.86	0.86	0.88	0.90
**S17**	5	0.81	0.005	0.80	0.80	0.81	0.81	0.81
**S22b**	5	0.84	0.018	0.82	0.83	0.84	0.86	0.86
**S25**	5	0.85	0.026	0.82	0.83	0.86	0.87	0.88
**S26**	5	0.83	0.019	0.80	0.83	0.83	0.84	0.85
**S27**	5	0.86	0.034	0.82	0.85	0.86	0.88	0.91
**S28**	5	0.88	0.018	0.87	0.87	0.87	0.89	0.91
**S35**	5	0.86	0.029	0.82	0.86	0.87	0.87	0.90
**S37**	5	0.82	0.011	0.80	0.81	0.82	0.82	0.83
**S41**	5	0.85	0.014	0.84	0.84	0.84	0.86	0.87
**S42**	5	0.82	0.011	0.80	0.82	0.82	0.82	0.83
**S44**	5	0.84	0.013	0.83	0.83	0.84	0.85	0.86
**S46**	5	0.85	0.011	0.84	0.85	0.85	0.86	0.87
**S50**	5	0.82	0.032	0.78	0.80	0.81	0.84	0.86
**S52**	5	0.86	0.017	0.83	0.85	0.86	0.87	0.87
**S53**	5	0.93	0.019	0.90	0.93	0.94	0.94	0.95
**S54**	10	0.87	0.019	0.85	0.85	0.87	0.89	0.90
**S57**	5	0.80	0.005	0.80	0.80	0.80	0.81	0.81
**S60**	5	0.86	0.016	0.84	0.85	0.85	0.87	0.88
**S63**	5	0.84	0.011	0.83	0.84	0.84	0.85	0.86
**S65**	5	0.90	0.011	0.88	0.90	0.90	0.90	0.91
**S66**	5	0.80	0.008	0.79	0.79	0.80	0.80	0.81
**S67**	5	0.83	0.015	0.81	0.83	0.84	0.84	0.85
**S68**	5	0.80	0.01	0.79	0.79	0.80	0.81	0.81
**S71**	5	0.86	0.043	0.82	0.84	0.85	0.88	0.93
**S72**	10	0.83	0.016	0.81	0.82	0.83	0.84	0.86

Commented [NT1]: Letter t was missing

Accession	n	Mean 2C value	S.D.	Min	Q1	Median	Q3	Max
**S75**	6	0.83	0.019	0.80	0.82	0.83	0.85	0.85
**S76**	5	0.82	0.007	0.81	0.82	0.82	0.82	0.83
**S79**	7	0.85	0.016	0.83	0.84	0.84	0.86	0.87
**S81**	7	0.84	0.017	0.81	0.84	0.85	0.85	0.86
**S82**	5	0.82	0.018	0.80	0.81	0.82	0.84	0.84
**S83**	5	0.84	0.015	0.82	0.83	0.84	0.84	0.86
**S84**	5	0.83	0.018	0.81	0.81	0.83	0.84	0.85
**S85**	5	0.87	0.019	0.85	0.86	0.88	0.88	0.90
**S86**	5	0.81	0.033	0.77	0.80	0.80	0.81	0.86
**S91**	6	0.80	0.022	0.77	0.78	0.80	0.81	0.83
**S92**	5	0.87	0.016	0.84	0.87	0.87	0.88	0.88
**S93**	5	0.81	0.009	0.81	0.81	0.81	0.81	0.83
**S94**	7	0.85	0.026	0.82	0.83	0.86	0.87	0.89
**S95**	5	0.87	0.029	0.85	0.85	0.86	0.88	0.92
**S96**	5	0.78	0.008	0.77	0.78	0.78	0.79	0.79
**S99**	5	0.84	0.013	0.83	0.83	0.84	0.85	0.86
**S100**	5	0.84	0.013	0.82	0.83	0.83	0.85	0.85
**S101**	5	0.88	0.022	0.85	0.87	0.88	0.88	0.91
**S104**	12	0.83	0.027	0.77	0.82	0.83	0.84	0.86
**S110**	5	0.87	0.022	0.85	0.86	0.86	0.89	0.90
**S111**	5	0.87	0.018	0.85	0.86	0.88	0.89	0.89
**S116**	5	0.90	0.019	0.89	0.89	0.89	0.92	0.93
**S118**	5	0.84	0.019	0.81	0.82	0.85	0.85	0.85
**S120**	5	0.85	0.036	0.79	0.86	0.86	0.87	0.88
**S122**	5	0.85	0.019	0.82	0.85	0.86	0.86	0.87
**S123**	5	0.82	0.007	0.81	0.82	0.82	0.82	0.83
**S125**	5	0.92	0.026	0.89	0.90	0.93	0.94	0.95
**S126**	5	0.88	0.013	0.86	0.88	0.89	0.89	0.89
**S130**	5	0.89	0.021	0.86	0.88	0.89	0.91	0.91
**S132**	5	0.95	0.031	0.92	0.93	0.94	0.95	1.00
**S133**	5	0.85	0.018	0.83	0.83	0.84	0.86	0.87
**S134**	5	0.86	0.022	0.83	0.85	0.87	0.88	0.88
**S135**	5	0.86	0.032	0.84	0.85	0.85	0.86	0.92
**S136**	6	0.86	0.017	0.84	0.85	0.86	0.87	0.89
**S137**	5	0.89	0.021	0.87	0.87	0.89	0.90	0.92
**S138**	10	0.87	0.013	0.85	0.86	0.87	0.88	0.89
**S139**	5	0.90	0.033	0.86	0.87	0.91	0.93	0.93
**S140**	5	0.90	0.033	0.85	0.90	0.90	0.92	0.94
**STh**[a]	8	0.73	0.01	0.72	0.72	0.72	0.74	0.74

[a]STh (*S indicum* from Thailand) was purchased on Market.

Table S2. Coefficients of variation (cf) of sesame genome size among accessions (All accessions) and within genetic (Gr1, Gr2 and Gr3) and agro-morphological (AgroM1, AgroM2 and AgroM3) groups.

Commented [NT2]: revised

		cf.
All accessions	+ STh	0.048
	- STh	0.044
Genetic Groups	Gr1	0.037
	Gr2	0.048
	Gr3	0.043
Agro-morphological Groups	AgroM1	0.045
	AgroM2	0.044
	AgroM3	0.042

Table S3. Pairwise comparisons of sample medians using Dunn's test among Niger sesame accessions. Only the 58 pairs of accessions with a significant difference are shown.

Pair Accessions	*Z*	*P*[1]
S12 - S91	4.339	0.039*
S12 - S96	4.528	0.016*
S13 - S15	4.683	0.008**
S13 - S17	4.511	0.018*
S13 - S57	4.578	0.013*
S13 - S66	4.722	0.006**
S13 - S68	4.656	0.009**
S13 - S91	4.807	0.004**
S13 - S96	4.975	0.002**
S15 - S53	-4.886	0.003**
S15 - S65	-4.411	0.028*
S17 - S53	-4.714	0.007**
S37 - S53	-4.334	0.04*
S53 - S57	4.78	0.005**
S53 - S66	4.925	0.002**
S53 - S68	4.859	0.003**
S53 - S86	4.345	0.038*
S53 - S91	5.018	0.001**
S53 - S93	4.417	0.027*
S53 - S96	5.178	0.001**
S57 - S65	-4.306	0.045*
S65 - S66	4.451	0.023*
S65 - S68	4.384	0.032*
S65 - S91	4.523	0.017*
S65 - S96	4.703	0.007**
S104 - S132	-4.546	0.015*
S104 - S53	-4.454	0.023*
S116 - S15	4.483	0.02*
S116 - S17	4.311	0.044*

S116 - S57	4.377	0.033*
S116 - S66	4.522	0.017*
S116 - S68	4.456	0.023*
S116 - S91	4.598	0.012*
S116 - S96	4.775	0.005**
S125 - S15	4.736	0.006**
S125 - S17	4.564	0.014*
S125 - S57	4.63	0.01*
S125 - S66	4.775	0.005**
S125 - S68	4.709	0.007**
S125 - S91	4.862	0.003**
S125 - S96	5.028	0.001**
S130 - S96	4.353	0.037*
S132 - S15	4.963	0.002**
S132 - S17	4.791	0.005**
S132 - S37	4.411	0.028*
S132 - S42	4.337	0.039*
S132 - S57	4.858	0.003**
S132 - S66	5.002	0.002**
S132 - S68	4.936	0.002**
S132 - S72	4.336	0.04*
S132 - S86	4.422	0.027*
S132 - S91	5.099	0.001**
S132 - S93	4.494	0.019*
S132 - S96	5.255	0.000***
S137 - S96	4.377	0.033*
S139 - S96	4.442	0.024*
S140 - S91	4.295	0.047*
S140 - S96	4.486	0.02*

[1]Adjusted p-value with Holm's correction. Statistical significance was assigned at $P < 0.05$ (*), $P < 0.01$ (**) and $P < 0.001$ (***).

Table S4. Pairwise comparisons of sample medians using Dunn's test among genetic (Gr1, Gr2 and Gr3)[21] and agro-morphological groups [22] (Table 2).

	**Groups**	***Z***	***P*[1]**
Genetic groups	Gr1-Gr2	1.41	0.159^{ns}
	Gr1-Gr3	1.79	0.145^{ns}
	Gr2-Gr3	-3.405	0.002**
Agro-morphological groups	AgroM1-AgroM2	2.497	0.025*
	AgroM1-AgroM3	-0.0493	0.622^{ns}
	AgroM2-AgroM3	-3.487	0.001**

[1]Adjusted p-value with Holm's correction.

Statistical significance was assigned at $P < 0.05$ (*), $P < 0.01$ (**) and $P < 0.001$ (***); ns: non-significant.

Table S5. Pairwise comparisons of sample medians using Dunn's test among genetic (Gr1, Gr2 and Gr3)[21], agro-morphological groups [22] and STh, the Tai sesame as a separate group (Table 2).

	**Groups**	***Z***	***P*[1]**
Genetic groups	Gr1-Gr2	1.382	0.167[ns]
	Gr1-Gr3	-1.759	0.157[ns]
	Gr2-Gr3	-3.339	0.003**
Genetic groups / Sth	Gr1-STh	4.612	0.000***
	Gr2-STh	4.1085.269	0.000***
	Gr3-STh		0.000***
Agro-morphological groups	AgroM1-AgroM2	2.448	0.029*
	AgroM1-AgroM3	-0.483	0.629[ns]
	AgroM2-AgroM3	-3.419	0.002**
Agro-morphological groups / STh	AgroM1-STh	4.877	0.000***
	AgroM2-STh	3.955	0.000***
	AgroM3-STh	5.154	0.000***

[1]Adjusted p-value with Holm's correction.

Statistical significance was assigned at $P < 0.05$ (*), $P < 0.01$ (**) and $P < 0.001$ (***); ns: non-significant.
